# Comparison Study of Two Fumonisin-Degrading Enzymes for Detoxification in Piglets

**DOI:** 10.3390/toxins16010003

**Published:** 2023-12-20

**Authors:** Zhenlong Wang, Zonghao Lv, Tibor Czabany, Veronika Nagl, Rudolf Krska, Xiumin Wang, Bing Han, Hui Tao, Jie Liu, Jinquan Wang

**Affiliations:** 1 Key Laboratory of Feed Biotechnology, Ministry of Agriculture and Rural Affairs, Institute of Feed Research, Chinese Academy of Agricultural Sciences, No. 12 Zhongguancun South Street, Beijing 100081, China; wangzhenlong02@caas.cn (Z.W.);; 2 Laboratory of Pet Nutrition and Food, Institute of Feed Research, Chinese Academy of Agricultural Sciences, No. 12 Zhongguancun South Street, Beijing 100081, China; 3 College of Animal Science and Technology, Hunan Agricultural University, No. 1 Furong District, Changsha 410128, China; 4 dsm-firmenich, Animal Nutrition and Health R&D Center, Technopark 1, 3430 Tulln, Austriaveronika.nagl@dsm.com (V.N.); 5 Department of Agrobiotechnology IFA-Tulln, Institute of Bioanalytics and Agro-Metabolomics, University of Natural Resources and Life Sciences, Vienna (BOKU), Konrad-Lorenz-Str. 20, 3430 Tulln, Austria; rudolf.krska@boku.ac.at; 6 Institute for Global Food Security, School of Biological Sciences, Queens University Belfast, University Road, Belfast BT7 1NN, UK; 7 Austrian Competence Centre for Feed and Food Quality, Safety & InnovationFFoQSI GmbH, Konrad-Lorenz-Str. 20, 3430 Tulln, Austria

**Keywords:** fumonisins, Sa/So, FumDSB, FUM*zyme*, degrading enzyme

## Abstract

Fumonisins (FBs), particularly fumonisin B1 (FB1) and fumonisin B2 (FB2) produced mainly by *Fusarium verticillioide* and *Fusarium proliferatum*, are common contaminants in animal feed and pose a serious threat to both animal and human health. The use of microbial enzymes to efficiently and specifically convert fumonisins into non-toxic or low-toxic metabolites has emerged as the most promising approach. However, most of the available enzymes have only been evaluated in vitro and lack systematic evaluation in vivo. In this study, the detoxification efficacy of two carboxylesterases, FumD (FUM*zyme*^®^) and FumDSB, was evaluated comparatively in piglets. The results show that feeding piglets 4.4 mg/kg FBs-contaminated diets for 32 days did not significantly affect the average daily gain, organ indices, and immunoglobulins of the piglets. However, a significant reduction (21.2%) in anti-inflammatory cytokine interleukin-4 was observed in the FBs group, and supplementation with FUM*zyme*^®^ and FumDSB significantly increased interleukin-4 by 62.1% and 28.0%, respectively. In addition, FBs-contaminated diets resulted in a 3-fold increase in the serum sphinganine/sphingosine (Sa/So) ratio, which is a specific biomarker that has been used to accurately reflect fumonisin levels. The serum Sa/So ratio was significantly reduced by 48.8% after the addition of FUM*zyme*^®^, and was insignificantly reduced by 8.2% in the FumDSB group. These results suggested that FUM*zyme* was more effective than FumDSB in mitigating FBs toxicity in piglets by down-regulating the Sa/So ratio.

## 1. Introduction

Fumonisins are mycotoxins produced by various species of fungi, primarily *Fusarium verticillioide* and *Fusarium proliferatum*. These toxins commonly contaminate maize (corn) and other cereal grains worldwide, posing significant risks to the livestock industry [1]. More than 20 species of fumonisins have been identified and classified into four categories (A, B, C, and P) based on their structural characteristics [2]. Of these, fumonisin B (FB), particularly fumonisin B1 (FB1) and fumonisin B2 (FB2), are the most prevalent and concerning mycotoxins [3], which can cause acute or chronic poisoning in animals and humans via the food chain [4]. The consumption of FBs-contaminated feed has been associated with various health issues in animals, including reduced growth, immune suppression, reproductive problems, and increased susceptibility to diseases [5,6,7]. Studies have shown that FBs can cause cerebral white matter softening disease in horses, with symptoms including dementia, motor dysfunction, and blindness [8,9]. Pigs are particularly susceptible to fumonisin toxicity, developing porcine pulmonary edema syndrome characterized by severe respiratory distress [10]. Epidemiological data show an association between FB1 contamination of the human diet and a high incidence of esophageal cancer [11]. The International Agency for Research on Cancer has classified FB1 as a class B carcinogen [12].

FBs usually have a special large branched structure consisting of 19–20 linear carbon skeletons with various functional groups distributed on both sides, which makes the entrance of FBs into the interlayer space of adsorbents difficult, and they are stable under UV light and high temperature. Traditional physical strategies are not effective in removing FBs from feed and even result in the loss of nutrients in feed [13,14]. In addition, the toxin-carrying adsorbents will cause environmental pollution after being excreted from the animals. Chemical methods may produce potentially toxic compounds in the detoxification process with safety hazards. Biological detoxification methods have also gained attention in recent years [15]. Scientists have identified and characterized several enzymes capable of degrading fumonisins, offering potential solutions for the detoxification of FBs-contaminated feed and agricultural products [15]. These enzymes can effectively cleave fumonisin molecules, producing less toxic compounds. The carboxylesterase FumD is generally known as the most extensively studied FBs degradation enzyme derived from *Sphingopyxis* sp. MTA144 [16,17]. FumD can catalyze the hydrolysis of FB1 into non-toxic hydrolyzed products and it was developed as a commercial fumonisin-degrading enzyme product FUM*zyme*^®^ by BIOMIN in 2014 [18]. FUM*zyme*^®^ is designed for use in animal feed to mitigate the toxic effects of fumonisins, thereby improving animal health and performance. Li et al. screened another carboxylesterase FumDSB from the genus *Sphingomonadales bacterium* from GenBank based on a sequence-similarity search using BLASTp analysis [19], and found that the addition of FumDSB can reduce the anorexia effects of FB1 by regulating several brain–gut peptides in both the hypothalamus and the jejunum of growing pigs [20].

Several studies have been conducted to evaluate the efficacy of fumonisin-degrading enzymes in animal models. These studies typically added enzyme products to the diet, followed by the evaluation of feed intake, growth performance, organ indices, and immune response after exposure to fumonisins. However, there is no global consensus on methods for assessing the in vivo effects of fumonisin-degrading enzymes. In 2012, the European Food Safety Authority established an evaluation system based on the utilization of mycotoxin biomarkers [21]. One of the most well-known exposure-based biomarkers is aflatoxin M1 (AFM1), which is the hydroxylation product of aflatoxin B1 (AFB1) by specific cytochrome P450 enzymes [22]. However, due to the low bioavailability of fumonisin (<6%) and its limited metabolism in mammals, the detection of FB1 and its metabolites in biological fluids often fails [23,24]. The most extensive research for fumonisin biomarkers has been that related to the disruption of de novo sphingolipid biosynthesis [25]. Due to its analogous structure to sphingomyelin bases, sphinganine (Sa) and sphingosine (So), FBs have been identified as potent inhibitors of sphingosine N-acyl transferase (ceramide synthase), which acylates the amino group of Sa with a fatty acid molecule to produce dihydroceramide [26]. Shephard et al. found that the elevation of the Sa/So ratio is associated with FBs exposure in livestock [23]. With the ingestion of FBs by livestock, it can disrupt sphingolipid metabolism and lead to the rapid accumulation of Sa, and, to a lesser extent So, in biological fluids and tissues, resulting in alterations in the Sa/So ratio.

In this study, we adopted the biomarker approach recommended by the European Food Safety Authority and selected weaned piglets with high susceptibility to fumonisins as test animals to evaluate the in vivo effects of two fumonisin-degrading enzymes: FUM*zyme*^®^ and FumDSB. We used the Sa/So ratio in serum as a specific biomarker and also monitored relevant non-specific indicators. This study provides a valuable reference for the development of a uniform protocol for the in vivo effectiveness of FBs-degrading enzymes.

## 2. Results

### 2.1. Effect of FUMzyme^®^ and FumDSB Supplementation on Growth Performance of Piglets Fed Fumonisin-Contaminated Diets

**Figure 1:**
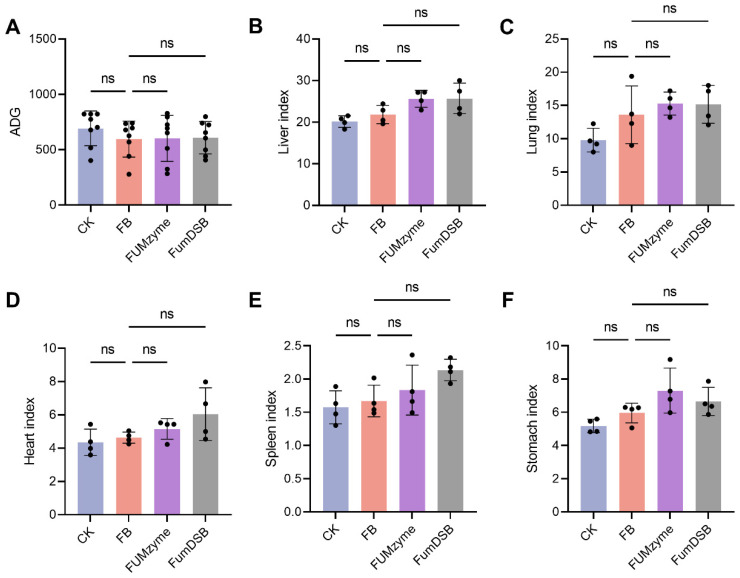
Effect of FBs, FUM*zyme*^®^, and FumDSB supplementation on growth performance and organ indices of piglets. (**A**) Average daily gain (ADG). (**B**) Liver index. (**C**) Lung index. (**D**) Heart index. (**E**) Spleen index. (**F**) Stomach index. CK, the basal diet group; FB, 4.4 mg/kg FBs-contaminated diets; FUM*zyme*, 4.4 mg/kg FBs-contaminated diets supplemented with 0.2% FUM*zyme*^®^; FumDSB, 4.4 mg/kg FBs-contaminated diets supplemented with 2% FumDSB. The results of (**A**) are given as the mean ± SD (*n* = 8), and (**B**–**F**) are given as the mean ± SD (*n* = 4). The “ns” indicates no significant difference between the two groups.

Throughout the 32-day feeding period, all piglets appeared healthy with no mortality. Compared with the basal diet group (CK group), no significant differences (*p* > 0.05) in average daily gain (ADG) were observed in the FB group (4.4 mg/kg FBs-contaminated diet), FUM*zyme*^®^ group, and FumDSB group (Figure 1A). Meanwhile, livers, lungs, hearts, spleens, and stomachs were collected from the piglets at the end of the trial to investigate the effects of FBs on these organs, and the results showed that the FBs-contaminated diets had no significant effect (*p* > 0.05) on the organ indices (Figure 1B–F). These results suggested that diets contaminated with 4.4 mg/kg of FBs had no significant adverse effects on ADG and the organ indices of piglets.

### 2.2. Effect of FUMzyme^®^ and FumDSB on Serum Biochemical Indices in Piglets

**Figure 2:**
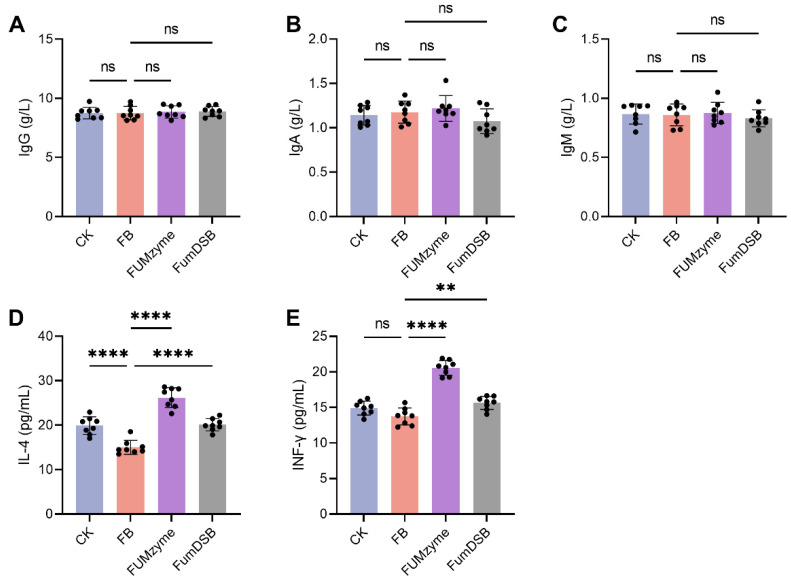
Effect of FBs*,* FUM*zyme*^®^, and FumDSB supplementation on immunity of piglets. (**A**) IgG. (**B**) IgA. (**C**) IgM. (**D**) IL-4. (**E**) INF-γ. CK, the basal diet group; FB, 4.4 mg/kg FBs-contaminated diets; FUM*zyme*, 4.4 mg/kg FBs-contaminated diets supplemented with 0.2% FUM*zyme*^®^; FumDSB, 4.4 mg/kg FBs-contaminated diets supplemented with 2% FumDSB. The results of (**A**–**E**) are presented as the mean ± SD (*n* = 8). The “ns” indicates no significant difference between the two groups, and asterisks indicate a significant difference between the two groups (** *p* < 0.01, **** *p* < 0.0001).

To analyze the effects of FUM*zyme*^®^ or FumDSB supplement on immunity and inflammation in piglets, Immunoglobulins (IgG, IgA and IgM) and cytokines (interleukin-4 (IL-4) and interferon-γ (INF-γ)) in piglet serum were detected after 32-day feeding period. As shown in Figure 2A–C, IgG, IgA, and IgM were not significantly different in any of the treatment groups (*p* > 0.05). However, a significant reduction (21.2%) in anti-inflammatory cytokine interleukin-4 (IL-4) was found in the FB group compared to the CK group (*p* < 0.0001), which indicated the occurrence of inflammation in the FB group, and the supplementation of FUM*zyme*^®^ and FumDSB significantly (*p* < 0.0001) promoted IL-4 levels by 62.1% and 28.0%, respectively (Figure 2D), suggesting that FUMzyme^®^ and FumDSB can alleviate FBs-induced inflammation by upregulating anti-inflammatory IL-4. INF-γ is a critical cytokine for innate and adaptive immunity against viral, some bacterial and toxic compounds, and FB treatment had no significant effect on the INF-γ level compared to the CK group (*p* > 0.05). However, FUM*zyme*^®^ and FumDSB supplementation significantly (*p* < 0.01) contributed to increases in INF-γ by 46.7% and 12.9%, respectively, compared to the FB group (Figure 2E), suggesting that FUM*zyme*^®^ and FumDSB supplementation may be associated with immunity in piglets.

### 2.3. Effect of FUMzyme^®^ and FumDSB on the Sa, So, and Sa/So Ratio in the Serum of Piglets

**Figure 3:**
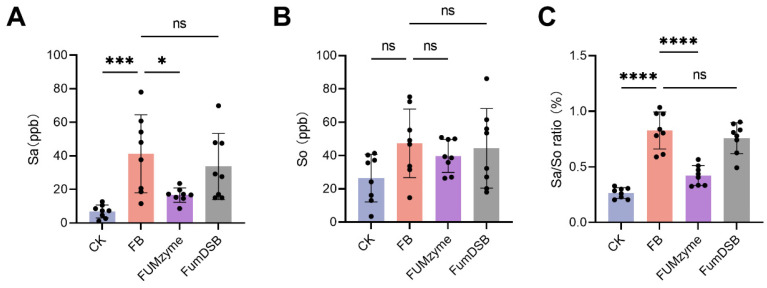
Effect of FBs, FUMzyme^®^, and FumDSB supplementation on sphingoid bases and their ratio. Serum concentration of (**A**) Sphinganine (Sa), (**B**) Sphingosine (So), and the respective calculated Sa/So ratio (**C**). CK, the basal diet group; FB, 4.4 mg/kg FBs-contaminated diets; FUM*zyme*, 4.4 mg/kg FBs-contaminated diets supplemented with 0.2% FUM*zyme*^®^; FumDSB, 4.4 mg/kg FBs-contaminated diets supplemented with 2% FumDSB. The results are given as the mean ± SD (*n* = 8). The “ns” indicates no significant difference between the two groups, and asterisks indicate a significant difference between the two groups (* *p* < 0.05, *** *p* < 0.001, **** *p* < 0.0001).

FBs is an inhibitor of sphinganine N-acyltransferase and can interfere with sphingolipid metabolism, resulting in an increased sphinganine (Sa) to sphingosine (So) ratio, which has been proposed as a biomarker of fumonisin exposure in animals [26]. At the end of the feeding trial, serum levels of Sa and So were measured and their ratio was calculated. As shown in Figure 3, serum Sa and Sa/So were significantly elevated in the FB group by 5.02- and 2.12-fold, respectively, compared to the animals receiving the basal diet (*p* < 0.001) (Figure 2A,C). In contrast, serum So levels were not significantly different across all groups (*p* > 0.05) (Figure 2B). Compared to the FB group, Sa and Sa/So levels in the FUM*zyme*^®^ group were significantly reduced by 59.7% (*p* < 0.05) and 48.8% (*p* < 0.0001), respectively. In the FumDSB group, the Sa and Sa/So ratios did not markedly decrease until the end of the experiment (*p* > 0.05). These results suggest that the addition of FUM*zyme*^®^ to the diet can significantly alleviate the abnormal elevation of Sa/So levels induced by FBs, whereas FumDSB did not have a significant Sa/So reducing effect.

## 3. Discussion

Although several biological enzymes capable of degrading fumonisins have been identified in recent years, most of them are still at the laboratory stage due to a low degradation efficiency and poor stability, and the only one that has been commercially applied as a feed additive is FUM*zyme*^®,^ approved by the European Food Safety Authority [18]. The enzyme component in FUM*zyme*^®^ is the fumonisin esterase FumD. Another esterase FumDSB was identified from *Sphingomonadales bacterium* and shared only 34.3% identity with the amino acid sequence of the carboxylesterase FumD [19]. In the present study, we aimed to investigate the detoxification effect of two fumonisin esterases on FBs-exposed piglets with a special emphasis on the biomarker Sa/So ratio.

A series of studies have shown that FBs have certain toxic effects in organs such as the liver, lungs, and heart in different animals [27]. In our study, there was no significant change in ADG and organ index at an FBs contamination concentration of 4.4 mg/kg (Figure 1A), whereas in previous reports a significantly lower ADG was found in 5.0 mg/kg-contaminated feed. This may be due to the different levels of FBs contamination, and a significant reduction in growth performance could be observed at high doses of FBs contamination. Similar to the study by Rao et al., a linear decrease in ADG occurred with increasing concentrations of FBs in the diet [28].

The immunotoxicity of FB1 has been accompanied by decreased immune responses [29]. Previous reports show that FB1 decreased IL-4 and increased IFN-γ synthesis at both the protein and mRNA levels [30]. In another report, Zhu et al. observed that the concentrations of IL-4 and IFN-γ in the cell culture supernatant were simultaneously decreased by FB1 treatment [31]. A similar result was found in our study: the decrease in IL-4 and increase in IFN-γ levels were observed upon FBs treatment compared to the CK group in vivo (Figure 2D). However, the concentration of INF-γ was not significantly changed by FBs (Figure 2E). These results indicated that IL4 is positively correlated with the level of FBs. Additionally, ingestion of the FBs-contaminated diets had no effect on the serum concentration of the immunoglobulin subset (IgG, IgA, and IgM), and this result was similar to a previous study that found that FB1 treatment had no effect on the concentration of these immunoglobulin subsets [30].

By inhibiting the key ceramide synthase, FBs disrupt the synthesis of sphingolipids, resulting in the accumulation of Sa and an increment in the Sa/So ratio over time. Therefore, it is suggested that the Sa/So ratio is the preferred biomarker to evaluate the efficacy of fumonisin-detoxifying feed additives. Dilkin et al. first reported a significant increase in the Sa/So ratio in the blood of weaned piglets after a single oral dose of FB1 (5 mg/kg of body weight) [32]. Furthermore, Masching et al. found that feeding FBs-contaminated diets leads to elevated Sa/So in piglet serum [16]. In our study, a similar increase in the Sa/So ratio was observed in the FBs group (Figure 3C). After supplementation of FUM*zyme*^®^ in the diet (FUM*zyme* group), a significant decrease (48.8%) in Sa/So occurred compared to the FBs group, indicating that FUM*zyme*^®^ has the potential to detoxify FBs in vivo. A gradual decrease (8.2%) in the Sa/So was observed in the FumDSB group (although not statistically significant; Figure 3C), which was not consistent with its high activity in vitro. Previous reports reported that FumDSB showed excellent FB1 degrading activity in buffer and porcine gastrointestinal fluids in vitro [16,19], suggesting that there is a large gulf between the in vitro results and the actual in vivo effects. Here are several conditions that may negatively impact the effect of FumDSB: (i) FumDSB may be degraded by some proteases present in the gastrointestinal tract; and (ii) the efficacy of FumDSB in detoxification was compromised due to insufficient concentration, making it inadequately potent for its intended purpose. In the future, improved stability through enzyme modification or dose–response studies is expected to address these issues.

It is worth noting that the concentration of FBs in the diet was 4.4 mg/kg in this study, which is less than the limit of 5 mg/kg specified in the Chinese Feed Hygiene Standard and is a relatively safe level. Although ADG and immune indicators did not change significantly with low-dose FBs contamination, Sa/So and interleukin-4 equivalents underwent a significant increase, suggesting that the biomarker Sa/So is more accurate in reflecting low-dose FBs contamination. In addition, the current results also suggest that low doses of FBs still cause abnormalities in piglets, which is similar to a previous report that dietary exposure to FBs at a low dose (3.7 mg/kg of feed) below the European Union (EU) regulatory limit also significantly increased the plasma Sa/So ratio and caused histological lesions in piglets [33]. Therefore, the current regulatory limit (both in China and the EU) is not sufficiently protective, especially for piglets, and the toxicity of low-dose FBs should be noted during the prevention and control of FBs contamination in animal husbandry.

In conclusion, data in this study aided us in evaluating the detoxification effect of two fumonisin-detoxifying enzymes (FumD and FumDSB) in piglets. Neither FumD (commercially available as FUM*zyme*^®^) nor FumDSB significantly affected the average daily gain, organ indices, and immunoglobulins of piglets fed 4.4 mg/kg FBs-contaminated diets. However, FUM*zyme*^®^ was able to significantly reduce (48.8%) the increase in the Sa/So ratio caused by fumonisins, while FumDSB showed a non-significant Sa/So reducing effect (8.2%). The present experiment provides clear evidence that FUM*zyme*^®^ is able to detoxify FBs in piglets, and the use of FUM*zyme*^®^ as a feed additive is a promising approach to control FBs contamination. Additionally, these results contribute to a deeper understanding of the use of biomarkers to evaluate the in vivo effects of fumonisin-degrading enzymes, especially for low doses of FBs that do not cause other significant changes in biochemical indices. Further studies should focus on the efficacy of different doses of enzyme supplementation for the detoxification of livestock and poultry consuming feed highly contaminated with FBs.

## 4. Methods

### 4.1. Source of Fumonisin Detoxification Enzymes FUMzyme^®^ and FumDSB

FUM*zyme*^®^ was obtained from dsm-firmenich (Tulln, Austria), which is produced from a genetically modified strain of *Komagataella pastoris*, and is already authorized by the European Commission for use as a feed additive in piglets. The fumonisin carboxylesterase FumDSB (Patent No: ZL201811171697.3) was expressed in *Escherichia coli* and provided by Dr. Zhongyuan Li of Tianjin University of Science and Technology. FumDSB consists of 511 amino acids and the theoretical molecular weight of pure FumDSB is 54 kDa. Li et al. reported that FB1 can be degraded by FumDSB to form HFB1 by releasing two tricarballylic acid groups.

### 4.2. Animal Feeding and Sampling

Animal experiments were approved by the Laboratory Animal Ethical Committee, Institute of Feed Research, Chinese Academy of Agricultural Sciences (AEC-CAAS-20161230 approved on 30 December 2016). A total of 32 piglets (Landrace × Yorkshire × Duroc, 20.44 ± 0. 54 kg BW, 60 ± 2 days old) were randomly divided into 4 groups. As shown in Table 1, the control group (CK) received a basal diet, and the FBs group was fed a basal diet contaminated with FB1 + FB2 (3841.7~4912.3 ppb). The FUM*zyme* and FumDSB groups were fed contaminated diets supplemented with 0.2% of FUM*zyme*^®^ (60 units/kg diet) or FumDSB enzymes, respectively. Piglets were fed and watered freely at 08:00 and 17:30 daily. The pre-test period was 7 days and the test period was 42 days. All piglets were fed a basal diet during the pre-test period to allow the animals to acclimate to the environment and to determine their feed intake. Deworming and vaccination were carried out according to routine procedures.

The nutrient levels of the basal diets were formulated according to NRC (1998) recommendations and are shown in Table 2. The fumonisin-contaminated diets were formulated using fumonisin-contaminated maize instead of the normal maize in the basal diets and were mixed proportionally with concentrates. Representative diet samples were taken to determine the mycotoxin levels. The sampling method was in accordance with the Feed Sampling Methods (GB/T 14699.1-1993). The levels of fumonisins (FB1 + FB2), zearalenone, aflatoxin B1, and deoxynivalenol in the diets were determined via liquid chromatography tandem–mass spectrometry (LC-MS) according to GB 13078-2017 in Romer Labs (Wuxi, China) (Table 1). Diets were prepared, bagged, and stored in a ventilated dry place.

### 4.3. Growth Performance and Organ Index

During the trial period, the weight of leftover feed was recorded daily. All piglets (*n* = 8) were weighed individually, and the average ADG values were calculated. Additionally, four piglets (*n* = 4) from each group were randomly selected for slaughter, and the liver, lung, kidney, heart, spleen, and stomach were quickly removed and weighed immediately after the removal of surface fat and other foreign bodies to calculate the organ index. Organ index (g/kg) = organ weight (g)/live pig weight (kg).

### 4.4. Measurement of Serum Biochemical Indicators

At the end of the positive test period, blood was collected fasting from the anterior vena cava of all piglets using vacuum blood collection tubes, and the blood samples (20 mL) were incubated at 37 °C for 30 min, transferred to a centrifuge at 168× *g* (3,000 × rpm) for 10 min, and the serum was separated. Immunoglobulin G, A, and M in serum were measured using the automatic biochemical analyzer (Huake ZY-1280, Beijing, China), and IFN-γ and IL-4 were measured using the porcine ELISA test kit (Beijing Jinhaikeou Biotechnology Co., Ltd., Beijing, China) according to the manufacturer’s instructions. Briefly, serial dilutions of recombinant porcine IL-4 and IFN-γ (0, 4, 8, 16, 32, 64 pg/mL) were added to a 96-well plate to construct a standard curve. After adding 10 μL sample and 100 μL of horseradish peroxidase (HRP)-conjugate reagent to each well, covering it with an adhesive strip and incubating it at 37 °C for 60 min, the absorbance of each well was measured at 450 nm after washing and 50 μL of stop solution (2 mol/L H_2_SO_4_) was added. The concentration of IL-4 and IFN-γ in the samples was then determined by comparing the OD_450_ of the samples to the standard curve.

### 4.5. HPLC-MS/MS Analysis of Serum Sa and So

The concentrations of serum Sa and So were determined using high-performance liquid chromatography–tandem mass spectrometry (HPLC-MS/MS). Frozen serum samples were thawed in a water bath (30 °C, 2 min). Thereafter, 600 µL of methanol/acetonitrile (*v*/*v*, 1:1) was added to 200 µL of serum sample, shaken for 30 min, and centrifuged at 20,000× *g* for 5 min. After the collection of the supernatant, the pellet was re-extracted with 300 µL of methanol/water (*v*/*v*, 80/20) for 30 s while vortexing and was subsequently centrifuged at 20,000× *g* for 5 min. Afterwards, supernatants were combined and dried at 30 °C. The extract was then re-dissolved in 600 µL of methanol/water (*v*/*v*, 80/20) and centrifuged at 20,000× *g* for 10 min. Finally, 300 µL was transferred into HPLC vials and subjected to HPLC-MS/MS analysis carried out on an Agilent 1290 Infinity II system (Agilent Technologies, Santa Clara, CA, USA) coupled to a QTrap 6500+ tandem mass spectrometer (Sciex).

The chromatographic separation (flow rate 500 µL/min, injection volume 2 µL) was achieved on a C18 Kinetex column (2.6 µm, 150 × 2.1 mm) projected by a pre-column (SecurityGuard ULTRA cartridge for C18 UHPLC, 2.1 mm, Phenomenex). Mobile phase A consisted of methanol/water/acetic acid (*v*/*v*/*v*, 40/59.8/0.2) and mobile phase B of methanol/acetic acid (*v*/*v*, 99.8/0.2). The gradient started at 65% B which was held for 1.7 min, increased to 100% (1.71 min), kept constant for 2.5 min, and then was finally reduced to 65% (2.51 min). Mass spectrometric detection of Sa and So was performed in selected reaction monitoring mode after electrospray ionization in positive mode, using the following parameters: Sa quantifier (quant) *m*/*z* 302.3/284.4, declustering potential (DP) 146 V, collision energy (CE) 19 V, collision cell exit potential (CXP) 14 V; Sa qualifier (qual) *m*/*z* 302.3/60.1, DP 146 V, CE 21 V, CXP 10 V; So quant *m*/*z* 300.3/282.4, DP 71 V, CE 15 V, CXP 6 V; So qual *m*/*z* 300.3/252.3, DP 71 V, CE 23 V, CXP 23 V. Serum samples of the feeding trial were worked up and analyzed in duplicate. Sa and So concentrations were determined on the basis of neat solvent calibration functions (MultiQuant 3.0.1 software, Sciex). Sa/So ratios were calculated.

### 4.6. Statistics

The test data were analyzed using the one-way ANOVA procedure of GraphPad Prism version 8 software (GraphPad Software, San Diego, CA, USA), and the significance of the differences was analyzed using Duncan’s multiple comparisons method, with *p* < 0.05 as the test level for the significance of each difference, and the results were expressed as mean ± standard deviation.

## Figures and Tables

**Table 1:** Designing animal groups.

Group	Content of Mycotoxin (mg/kg, ppm)				Enzyme Additives (%)
	FB1 + FB2	Zearalenone	Aflatoxin B1	Deoxynivalenol	
CK	0.62 ± 0.3	0.02 ± 0.001	<0.5	<10	-
FBs	4.41 ± 0.5	0.05 ± 0.015	0.004 ± 0.001	<10	-
FUM*zyme*^®^	4.41 ± 0.5	0.05 ± 0.015	0.004 ± 0.001	<10	0.2
FumDSB	4.41 ± 0.5	0.05 ± 0.015	0.004 ± 0.001	<10	0.2

**Table 2:** The ingredients and nutritional components of the basal diet.

Ingredients ^a^	%	Nutrition Levels	
Maize	30.00	DE/(MJ·kg^−1^)	12.79
Concentrate	70.00	Crude protein (%)	17.40
		Calcium (%)	0.78
		Total phosphorus (%)	0.52
		Lysine (%)	0.96
		Methionine + cysteine (%)	0.60

^a^ Provided per kg of diet: vitamin A, 12,000 IU; vitamin D3, 2000 IU; vitamin E, 40 IU; vitamin K3, 1.0 mg; vitamin B1, 1.0 mg; vitamin B2, 3.7 mg; vitamin B6, 3 mg; vitamin B12 0.02 mg; niacin,15 mg; folic acid, 0.6 mg; D-pantothenic acid, 10 mg; bilineurine, 250 mg; Mn, 40 mg; Fe, 100 mg; Zn, 100 mg; Cu, 180 mg; I, 0.30 mg; Co, 1.0 mg; Se, 0.30 mg.

## Data Availability

The data that support the findings of this study are available from the corresponding author upon reasonable request.

## References

[1] Voss K.A., Chamberlain W.J., Riley R.T., Bacon C.W., Norred W.P. (2009). In vitro and in vivo effects of fumonisins: Toxicity and mechanism of action. JSM Mycotoxins.

[2] Rheeder, John P., Marasas, Walter F.O., Vismer, Hester F. (2002). Production of fumonisin analogs by *Fusarium* species. Appl. Environ. Microbiol..

[3] Kamle M., Mahato D.K., Devi S., Lee K.E., Kang S.G., Kumar P. (2019). Fumonisins: Impact on agriculture, food, and human health and their management strategies. Toxins.

[4] Yang C., Song G., Lim W. (2020). Effects of mycotoxin-contaminated feed on farm animals. J. Hazard. Mater..

[5] Li X., Cao C., Zhu X., Li X., Wang K. (2020). Fumonisins B1 exposure triggers intestinal tract injury via activating nuclear xenobiotic receptors and attracting inflammation response. Environ. Pollut..

[6] Schatzmayr G., Streit E. (2013). Global occurrence of mycotoxins in the food and feed chain: Facts and figures. World Mycotoxin J..

[7] Gbore F.A. (2010). Growth performance and puberty attainment in growing pigs fed dietary fumonisin B(1). J. Anim. Physiol. Anim. Nutr..

[8] Constable P.D., Riley R.T., Waggoner A.L., Hsiao S.H., Haschek W.M. Serum sphingosine-1-phosphate and sphinganine-1-phosphate are elevated in horses exposed to fumonisin B1. In *AOAC International Midwest Section Final Program*; 2005; pp. 63–64. https://www.researchgate.net/publication/286418060_Serum_Sphingosine-1-Phosphate_and_Sphinganine-1-Phosphate_Are_Elevated_in_Horses_Exposed_to_Fumonisin_B1.

[9] Jovanovic M., Nesic S., Marinkovic D., Kukolj V., Trailovic D. (2015). Fumonisin toxicosis in horses. J. Comp. Pathol..

[10] Haschek W.M., Gumprecht L.A., Smith G., Tumbleson M.E., Constable P.D. (2001). Fumonisin toxicosis in swine: An overview of porcine pulmonary edema and current perspectives. Environ. Health Perspect..

[11] Xietong-Xin Z.H. (1998). Contamination of fungi and mycotoxins in foodstuffs in high risk area of esophageal cancer. Biomed. Environ. Sci..

[12] IARC Working Group on the Evaluation of Carcinogenic Risks to Humans (2002). Some traditional herbal medicines, some mycotoxins, naphthalene and styrene. IARC Monogr. Eval. Carcinog. Risks Hum..

[13] Liu Y., Tan L.P., Xing F.G., Chen Z.J. (2010). Reduction of fumonisins in maize using extrusion-cooking and nixtamalization method. Sci. Technol. Food Ind..

[14] Zhang J., Tang X., Cai Y., Zhou W.W. (2023). Mycotoxin contamination status of cereals in China and potential microbial decontamination methods. Metabolites.

[15] Qu L., Wang L., Ji H., Fang Y., Lei P., Zhang X., Jin L., Sun D., Dong H. (2022). Toxic mechanism and biological detoxification of fumonisins. Toxins.

[16] Masching S., Naehrer K., Schwartz-Zimmermann H.E., Sărăndan M., Schaumberger S., Dohnal I., Nagl V., Schatzmayr D. (2016). Gastrointestinal degradation of fumonisin B₁ by carboxylesterase FumD prevents fumonisin induced alteration of sphingolipid metabolism in Turkey and swine. Toxins.

[17] Heinl S., Hartinger D., Thamhesl M., Vekiru E., Krska R., Schatzmayr G., Moll W.D., Grabherr R. (2010). Degradation of fumonisin B1 by the consecutive action of two bacterial enzymes. J. Biotechnol..

[18] Bampidis V., Azimonti G., Bastos M.L., Christensen H., Dusemund B., Kos Durjava M., Kouba M., López-Alonso M., López Puente S., Marcon F. (2020). Safety and efficacy of fumonisin esterase from *Komagataella phaffii* DSM 32159 as a feed additive for all animal species. EFSA J..

[19] Li Z., Wang Y., Liu Z., Jin S., Pan K., Liu H., Liu T., Li X., Zhang C., Luo X. (2021). Biological detoxification of fumonisin by a novel carboxylesterase from *Sphingomonadales bacterium* and its biochemical characterization. Int. J. Biol. Macromol..

[20] Liu Q., Li F., Huang L., Chen W., Li Z., Wang C. (2021). FumDSB can reduce the toxic effects of fumonisin B(1) by regulating several brain-gut peptides in both the hypothalamus and jejunum of growing pigs. Toxins.

[21] EFSA Panel on Additives and Products or Substances used in Animal Feed (FEEDAP) (2012). Guidance for the preparation of dossiers for technological additives. EFSA J..

[22] Jager A.V., Tonin F.G., Baptista G.Z., Souto P.C., Oliveira C.A. (2016). Assessment of aflatoxin exposure using serum and urinary biomarkers in Sao Paulo, Brazil: A pilot study. Int. J. Hyg. Environ. Health.

[23] Shephard G.S., Westhuizen L.V.D., Sewram V. (2007). Biomarkers of exposure to fumonisin mycotoxins: A review. Food Addit. Contam..

[24] Voss K.A., Smith G.W., Haschek W.M. (2007). Fumonisins: Toxicokinetics, mechanism of action and toxicity. Anim. Feed Sci. Technol..

[25] Riley R.T., Merrill A.H. (2019). Ceramide synthase inhibition by fumonisins: A perfect storm of perturbed sphingolipid metabolism, signaling, and disease. J. Lipid Res..

[26] Knutsen H.K., Alexander J., Barregrd L., Bignami M., Brüschweiler B., Ceccatelli S., Cottrill B., Dinovi M., Edler L., Grasl-Kraupp B. (2018). Risks for animal health related to the presence of fumonisins, their modified forms and hidden forms in feed. EFSA J..

[27] Chen J., Wen J., Tang Y., Shi J., Mu G., Yan R., Cai J., Long M. (2021). Research progress on fumonisin B1 contamination and toxicity: A review. Molecules.

[28] Rao Z.X., Tokach M.D., Woodworth J.C., Derouchey J.M., Dritz S.S. (2020). Effects of fumonisin-contaminated corn on growth performance of 9 to 28 kg nursery pigs. Toxins.

[29] Li Y.C., Ledoux D.R., Bermudez A.J., Fritsche K.L., Rottinghaus G.E. (2000). The individual and combined effects of fumonisin B1 and moniliformin on performance and selected immune parameters in turkey poults. Poult. Sci..

[30] Taranu I., Marin D.E., Bouhet S., Pascale F., Bailly J.D., Miller J.D., Pinton P., Oswald I.P. (2005). Mycotoxin fumonisin B1 alters the cytokine profile and decreases the vaccinal antibody titer in pigs. Toxicol. Sci..

[31] Zhu F., Wang Y. (2022). Fumonisin B(1) induces immunotoxicity and apoptosis of chicken splenic lymphocytes. Front. Vet. Sci..

[32] Dilkin P., Direito G., Simas M.M.S., Mallmann C.A., Corrêa B. (2010). Toxicokinetics and toxicological effects of single oral dose of fumonisin B1 containing *Fusarium verticillioides* culture material in weaned piglets. Chem.-Biol. Interact..

[33] Terciolo C., Bracarense A.P., Souto P.C.M.C., Cossalter A.M., Dopavogui L., Loiseau N., Oliveira C.A.F., Pinton P., Oswald I.P. (2019). Fumonisins at doses below EU regulatory limits induce histological alterations in piglets. Toxins.

